# Acute retinal necrosis. Management and visual outcomes: a case series

**DOI:** 10.1186/s40942-022-00417-w

**Published:** 2022-09-15

**Authors:** Alireza Mojarrad, Arash Omidtabrizi, Mohammadreza Ansari Astaneh, Elham Bakhtiari, Elham Shiezadeh, Mohadeseh Hassani, Seyedeh Maryam Hosseini

**Affiliations:** 1 Eye Research Center, Mashhad University of Medical Sciences, Mashhad, Iran; 2 Retina Research Center, Mashhad University of Medical Sciences, Mashhad, Iran; 3 Khatam Al Anbia Eye Hospital, Abutalib crossroad, Ghareni Blvd, Mashhad, Iran

**Keywords:** Acute retinal necrosis, Retinal detachment, Anti viral, Intravitreal injection, Ganciclovir, Polymerase chain reaction

## Abstract

**Background:**

The present study reports the functional and anatomical outcomes of eyes with acute retinal necrosis(ARN).

**Methods:**

This is a retrospective case series conducted at a tertiary Eye Hospital from March 2015 to March 2020. Medical records of patients with clinical and laboratorial—Polymerase Chain Reaction (PCR)—diagnosis of ARN were reviewed. To identify factors related to the outcomes of visual acuity(VA) and retinal detachment (RD) over time, Cox proportional hazards regression modeling and survival analyses were used.

**Results:**

Twenty-three eyes of 23 patients (16 male, 7 female) were reviewed. Based on the PCR results, 16 cases (69.6%) had Varicella zoster virus, 3 cases (13%) had Cytomegalovirus, 1 patient (4.3%) had Herpes simplex virus associated ARN, and 1 case (4.3%) had negative PCR. The incident rate for ≥ 2-line VA gain was 0.28/eye-year (EY) (95% CI 0.21 ± 0.26) while the rate of severe vision loss was 0.09/eye-year (95% CI 0.05 ± 0.08). The RD development was observed at a rate of 0.43/eye-year (0.42 ± 0.02), which occurred in 9 eyes with a mean time of 100 days after the initial presentation of ARN. Patients’ age was the only factor associated with 2-line or more gain in VA over time with a hazard ratio of 0.921 (95% CI 0.854–0.993, P = 0.032).

**Conclusions:**

Generally, although being crucial, treatment is not highly effective in improvement of VA and decrease of RD development, as well as vision loss, in patients with ARN. However, treatment prevents fellow eye involvement efficiently. Younger age is associated with better response to treatment and more chance to achieve better VA.

## Background

First described by Urayama et al. in 1971, acute retinal necrosis (ARN) is a syndrome of acute Panuveitis with retinal periarteritis progressing to diffuse necrotizing retinitis and retinal detachment (RD) [1]. In 1982, Culbertson et al. found the Herpes virus in all layers of the affected retina by Electron microscopy, which confirmed the role of viral agents in this syndrome [2].

Contributive pathogens include Varicella zoster virus (VZV), followed by Herpes simplex virus (HSV)-1 and HSV-2, Cytomegalovirus (CMV), and Epstein Barr virus [3–5].

The ARN is a relatively rare condition, and as described in two national research projects in the UK, its annual incidence was estimated approximately at 0.5 to 0.63 new cases per million population [5, 6].

In 1994, the American Uveitis Society determined clinical diagnostic criteria for ARN (6) without the need for Polymerase chain reaction (PCR) testing of ocular fluid [7]. The PCR is useful in supporting a clinical diagnosis of ARN; however, treatment should not be postponed until PCR results are available [8].

Visual prognosis is generally unfavorable in ARN, and 48% of the affected eyes end up with 20/200 Snellen acuity or less after 6 months [6]. The most common cause of vision loss is RD which occurs in 20% to 73% of the affected eyes [4, 9]. Other possibly less disastrous causes of vision loss include chronic vitritis, epiretinal membrane, macular ischemia, macular edema, and optic neuropathy [10–12]. Bilateral disease and early or delayed contralateral eye involvement are important features of ARN with a significantly more incidence rate in untreated cases [13, 14].

Although there is no single guideline for ARN, the mainstay of treatment is high-dose systemic antiviral therapy with or without intravitreal adjuvant antiviral, followed by long-term oral prophylaxis. This regimen halts disease progression in the affected eye while preventing the involvement of the fellow eye [8, 15, 16]. Adding intravitreal antivirals has been investigated in several studies and has showed promise [17, 18].

Prophylactic laser retinopexy is another adjunctive therapy that has been widely studied, yet there is great controversy about the usefulness of laser. Patients with dense vitritis, which preclude laser treatment, often have more severe disease and develop RD, compared to milder cases with more clear media [9, 19, 20].

Although early vitrectomy seems an attractive option to reduce the risk of late RD onset, it is difficult to conclude its positive efficacy due to the discrepancy of baseline characteristics and follow-up periods between studies in this context, as well as the lack of a clear methodology [21–23].

Many studies in the literature investigating ARN diagnosis and treatment outcomes are relatively short-term reports; however, it is clear that complications of ARN, such as RD and contralateral involvement, would occur long after the initial disease. This study reports the long-term anatomic and visual outcomes up to 5 years after the manifestation of ARN.

## Methods

This study was a retrospective case series conducted at Khatam-Al-Anbia Eye Hospital (Mashhad, Iran) over 5 years from March 2015 to March 2020. The Ethics Committee of the Mashhad University of Medical Sciences (Mashhad, Iran) approved the study protocol, which also adhered to the tenets of the Declaration of Helsinki. Informed consent was also obtained from every patient before reviewing the records.

All patients met the criteria for ARN defined by the American Uveitis Society (6) and underwent anterior chamber paracentesis or vitreous tap for PCR testing.

### Demographic and clinical information assessment

Medical records of all the patients admitted to the hospital diagnosed with ARN and at least 6 months follow up were retrospectively reviewed, and data were collected about the signs and symptoms at presentation, immune system status, severity of the disease based on the grade of vitritis, the extent of retinal involvement (the number of retinal quadrants involved based on fundus photography and/or retinal drawing), the details of treatment strategy and adjuvant treatments, clinical course, VA outcome, the occurrence of RD, as well as demographic characteristics.

The inclusion criteria of the records to be analyzed were: definite clinical and laboratory diagnosis (except for 3 cases with only clinical diagnosis with good response to antiviral therapy) and a minimum of 6 month follow up period from the date of diagnosis. The exclusion criteria were: any previous history of intraocular inflammation, retinal detachment, or macula disease and poor adherence to treatment during the follow up period (if it was recorded in the patient’s file).

The Snellen VA data were converted into the logarithm of the minimum angle of resolution (LogMAR) units using the technique described by Holladay [24].

For patients who were unable to read the Snellen VA chart, VA was assigned based on the following criteria: counting fingers, 2.0 LogMAR VA, hand movements, and 3.0 LogMAR VA. Light perception and no-light perception were also noted but excluded from VA calculation.

### Antiviral therapy

According to the approved treatment protocol of the hospital, all patients received induction therapy with 10 mg/kg of systemic intravenous acyclovir three times a day or 3 g of valacyclovir orally in three divided doses for 7–10 consecutive days in combination with 2.0 mg/0.1 ml of intravitreal ganciclovir injection. Patients received intravitreal injections twice a week as the induction treatment until retinitis stabilized, at which time the frequency decreased to once a week until retinitis was considered inactive. The antiviral medication dose was adjusted for patients with evidence of renal insufficiency. Maintenance antiviral therapy began upon discharge with 800 mg of oral acyclovir twice a day or 1,000 mg of valacyclovir twice a day for at least 4 months.

### Corticosteroid therapy

At this stage, 1 mg/kg of oral prednisolone was administered after 48–72 h of the initiation of antiviral treatment and tapered during an average of 1 month after discharge from the hospital based on clinical response.

### Laser retinopexy

Laser retinopexy was performed for patients with relatively large areas of retinitis (more than three-disc areas) if they had enough space for putting 2–3 lines of laser spots in the adjacent healthy retina distant from macula as far as safe, no RD, and clear enough media to perform laser therapy. A barrier laser would be added during the follow-up if media clarity allowed.

### Pars plana vitrectomy

Pars plana vitrectomy was performed on refractory cases with dense vitritis precluding retinal examination, who were unresponsive to the combined systemic and intravitreal antiviral therapy, as well as corticosteroid, and also on patients who developed RD. In this study, early vitrectomy was not the approved protocol in milder cases with visible media allowing laser retinopexy.

### Statistical analysis

The IBM SPSS software (International Business Machines Corporation, Armonk, NY) was used for descriptive and inferential statistical analyses. Demographic and treatment information were summarized as frequencies.

Incidence rates of the major assessed outcomes were expressed as event rates per eye-year, defined as:

12 months of follow-up time for a single eye, to account for differential follow-up times. These main outcomes include 2-line or more VA gain, SVL of 20/200 or worse, and developing RD. To ascertain whether the outcome had been achieved, patients were required to maintain the level of VA gain or loss for two consecutive visits.

The VA at initial, 1-month, 3-month, 6-month, 12-month, and final time points were compared using a 2-tailed Paired Sample T-Test.

## Results

This study analyzed 23 eyes of 23 patients. Table 1 illustrates the demographic and baseline clinical characteristics of patients. Four patients out of 23 (17.4%) were immunosuppressed (one patient with leukemia, one patient with kidney transplantation, one patient with a history of gastric cancer, and one patient with a history of breast cancer) and three patients had a history of previous Herpes virus-associated illness (varicella, shingles, and HSV keratitis). None of the patients (including CMV retinitis cases) was HIV positive. All patients had unilateral involvement at presentation, and 18 (78.3%) had right eye involvement. None of the fellow eyes developed ARN during the whole follow-up period.

**Table 1:** Baseline characteristics of the study patients

Mean Age (Years) ± Standard Deviation	50.57 ± 13.658
Gender N (%)	
Male	16 (69.6%)
Female	7 (30.4%)
Laterality N (%)	
Right Eye	18 (78.3%)
Left Eye	5 (21.7%)
Baseline Visual Acuity In LogMA (Mean ± SD)	1.34 ± 0.95
Mean follow-up duration, month (Range)	11 (6–41)
Extent of retinitis N (%)	
1 Quadrant	0
2 Quadrants	9 (39.1%)
3 Quadrants	8 (34.8%)
4 Quadrants	6 (26.1%)
Type of anterior chamber reaction N (%)	
Granulomatous	12 (52.2%)
Non-Granulomatous	11 (47.8%)
PCR Result N (%)	
Varicella Zoster Virus	16 (69.6%)
Cytomegalovirus	3 (13.0%)
Herpes Simplex Virus (HSV)	1 (4.3%)
Negative	1 (4.3%)
No Data	2 (8.7%)
Treatment modality	
Intravenous acyclovir, N (%)	20 (86.9%)
Oral acyclovir, N (%)	4 (17.4%)
Oral valacyclovir, N (%)	14 (61%)
Oral valganciclovir, N (%)	3 (13%)
Intravitreal ganciclovir, N (%)	23 (100%)
Laser Retinopexy, N (%)	15 (65.2%)

logMAR: logarithm of the minimum angle of resolution; SD:  Standard Deviation

Samples of anterior chamber paracentesis or vitreous tap were collected from 21 eyes of 23 patients (14 males and 7 females), and the viral DNA was confirmed by PCR in 20 out of 21 patients (95%).

All patients were treated with systemic antiviral therapy, including acyclovir, valacyclovir, valganciclovir, or ganciclovir based on viral etiology and medication availability.

All patients received intravitreal ganciclovir. Totally, 65 intravitreal injections were performed with a median of three injections per eye (range 1–6 injections).

The mean time between the initiation of symptoms and the treatment was 22.14 ± 30.68 days.

Twelve eyes (52.2%) had granulomatous, and 11 eyes had non-granulomatous (47.8%) anterior chamber reaction. The type of uveitis (granulomatous or non-granulomatous) did not significantly affect the occurrence of rhegmatogenous retinal detachment (RRD) and the final VA outcome.

### Visual acuity outcomes

The mean initial va was 1.34 ± 0.95 LogMAR (Snellen equivalent, 20/438).

Thirteen eyes (56.5%) had an initial VA of < 20/200, nine eyes (39.1%) had an initial VA of 20/200-20/40, and one eye (4.3%) had an initial VA of > 20/40.

The distribution of retinitis encompassed two quadrants in nine eyes (39.1%), three quadrants in eight eyes (34.8%), and four quadrants in six eyes (26.1%).

The mean final VA was 1.14 ± 1.02 log MAR (Snellen equivalent, 20/276), with a mean follow-up time of 11 months (range 6–41 months).

Although the mean final VA improved with the treatment, it was not significantly better than the initial VA (P = 0.078).

Most VA improvement occurred within the first 6 months after the treatment (0.75 ± 0.95 LogMAR, Snellen equivalent 20/112). This improvement was not statistically significant (P = 0.075).

The incident rate of 2-line or more VA improvement was 0.28 events/eye-year (Table 2). Six eyes (26.1%) had 2-line or more VA improvement during the follow-up period.

**Table 2:** Incidence rate for visual acuity and retinal detachment outcomes

Outcome	Event rate per eye-year (95% confidence interval)
2-line or more visual acuity improvement	0.28 (0.21 ± 0.26)
Severe visual loss of 20/200 or worse	0.09 (0.05 ± 0.08)
Retinal detachment	0.43 (0.42 ± 0.02)

The incident rate of SVL of 20/200 or worse was 0.09 event/eye-year. Out of the 23 eyes with ARN, 2 (8.7%) had SVL of 20/200 or worse during the follow-up period.

### Retinal detachment outcomes

The incidence rate of RD was 0.43 events/eye-year. Out of the 23 eyes with ARN, 9 (39%) developed RD during the follow-up period.

The RD occurred at a mean of 100 ± 87 days (24–307 days) after the initial presentation of ARN.

Eyes with RD had a significantly worse initial LogMAR VA (P = 0.009) and final LogMAR VA (P = 0.003), compared to eyes without RD (14 eyes).

To account for the effect of these variables over time, the authors performed a Cox proportional hazards model analysis to assess these potential contributing factors.

The results showed that the patients’ age was a factor associated with 2-line or more gain in VA with a hazard ratio of 0.921 (95% CI 0.854–0.993, P = 0.032) in that younger patients had a higher possibility for visual improvement.

Other factors were not significantly associated with 2-line or more gain in VA and SVL (P > 0.05), which include the initial LogMAR VA, gender, RD, the extent of retinitis patches (the number of retinitis quadrants), and the number of intravitreal ganciclovir injections. Additionally, the initial LogMAR VA, gender, the extent of retinitis patches (the number of retinitis quadrants), and the number of intravitreal ganciclovir injections did not have a significant association with RD (P > 0.05).

The RD occurred in less than 3 months from the initiation of clinical presentation in six out of nine eyes and 4–10 months in three other patients.

The distribution of ARN lesions in eyes with RD was two quadrants in two eyes (22%), three quadrants in five eyes (56%), and four quadrant involvements in two eyes (22%).

The number of retinal quadrant involvements was not significantly different (P = 0.516) between eyes with and without RD.

The occurrence of subsequent RD was not statistically different in patients who received prophylactic laser therapy, compared to the non-laser group (P = 0.633). The final LogMAR VA significantly improved in eyes without RD (P = 0.04) whereas improvement in eyes with RD was not significant (P = 0.290).

### PCR results

The rate of positive PCR tests was 95.2% (20/21). The most common causative agent was VZV (16 eyes, 69.6%), followed by CMV (three eyes, 13%) and HSV (1 eye, 4.3%). In one case, PCR was negative (4.3%), yet the clinical presentation and the response to treatment confirmed the diagnosis. In two eyes (8.7%), the results were not available.

The number of HSV cases was not enough for analysis; therefore, the statistical comparison was made between causative agents VZV and CMV.

Patients with CMV ARN were older than VZV cases (mean age of 60 ± 4 years in VZV, compared to 50 ± 9 years in CMV cases); however, it was not statistically significant (P = 0.101).

In total, 25% of VZV patients were female while all CMV ARN cases were females (100%), and these proportions had significant associations (P = 0.036, Fisher’s Exact Test).

The rate of VA gain of 2-line or more in VZV patients (0.35 events/ eye-year) was higher than that in CMV patients with no event/eye-year. However, the difference was not statistically significant (P > 0.05).

The rate of RD was 0.56 event/eye-year in VZV cases and 0.20 event\EY in CMV cases (P > 0.05).

The rate of SVL was 0.07 events/eye-year in VZV and no event\eye-year in CMV patients (P > 0.05).

**Fig. 1:**
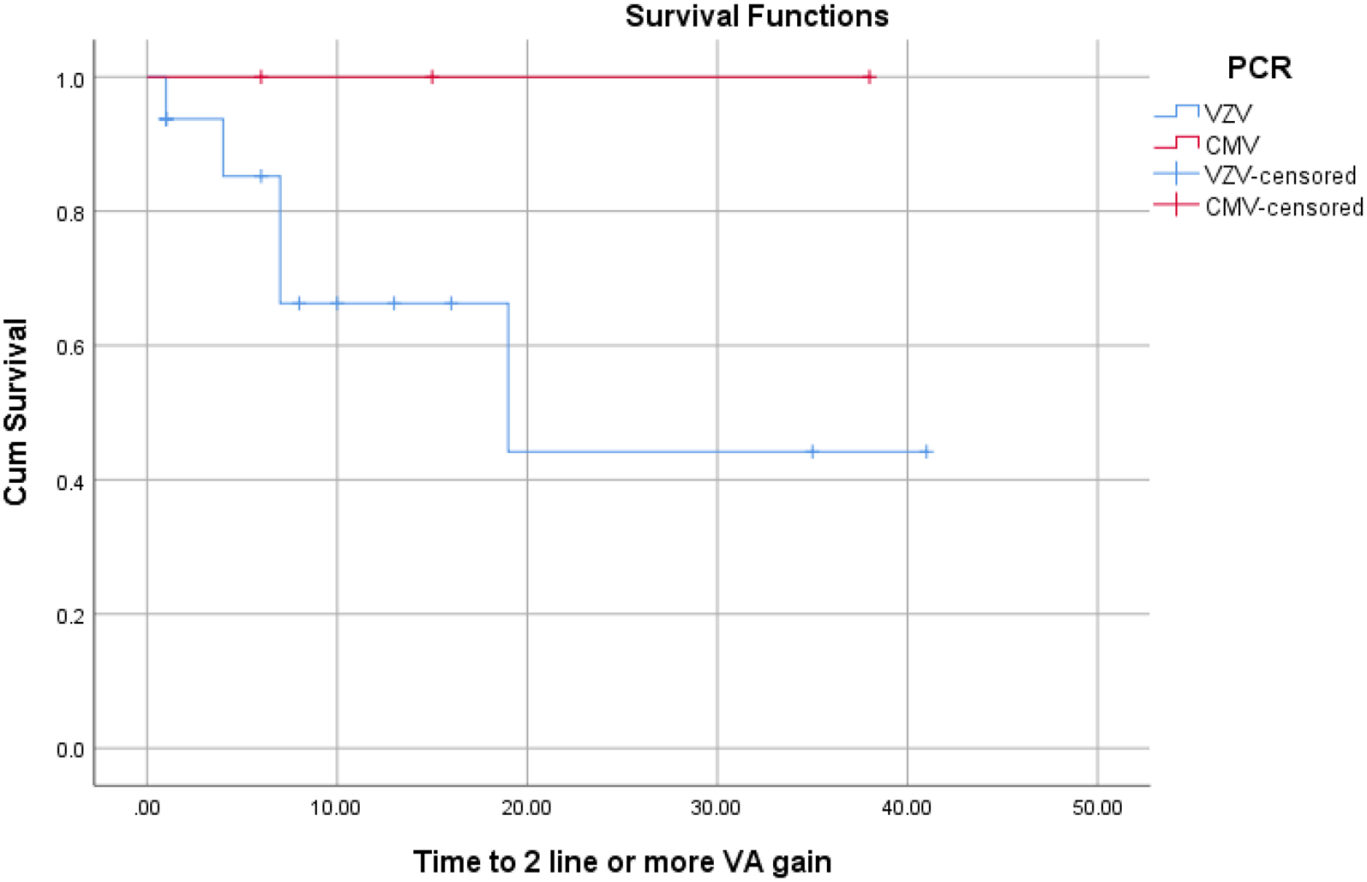
The rate of 2 lines or more VA gain over time (per month). Kaplan–Meier analysis showed a higher, but not statistically significant, rate in VZV group. Every fall (downward step) in the Blue line means one event (2 lines VA gain). No event happened in the CMV group

**Fig. 2:**
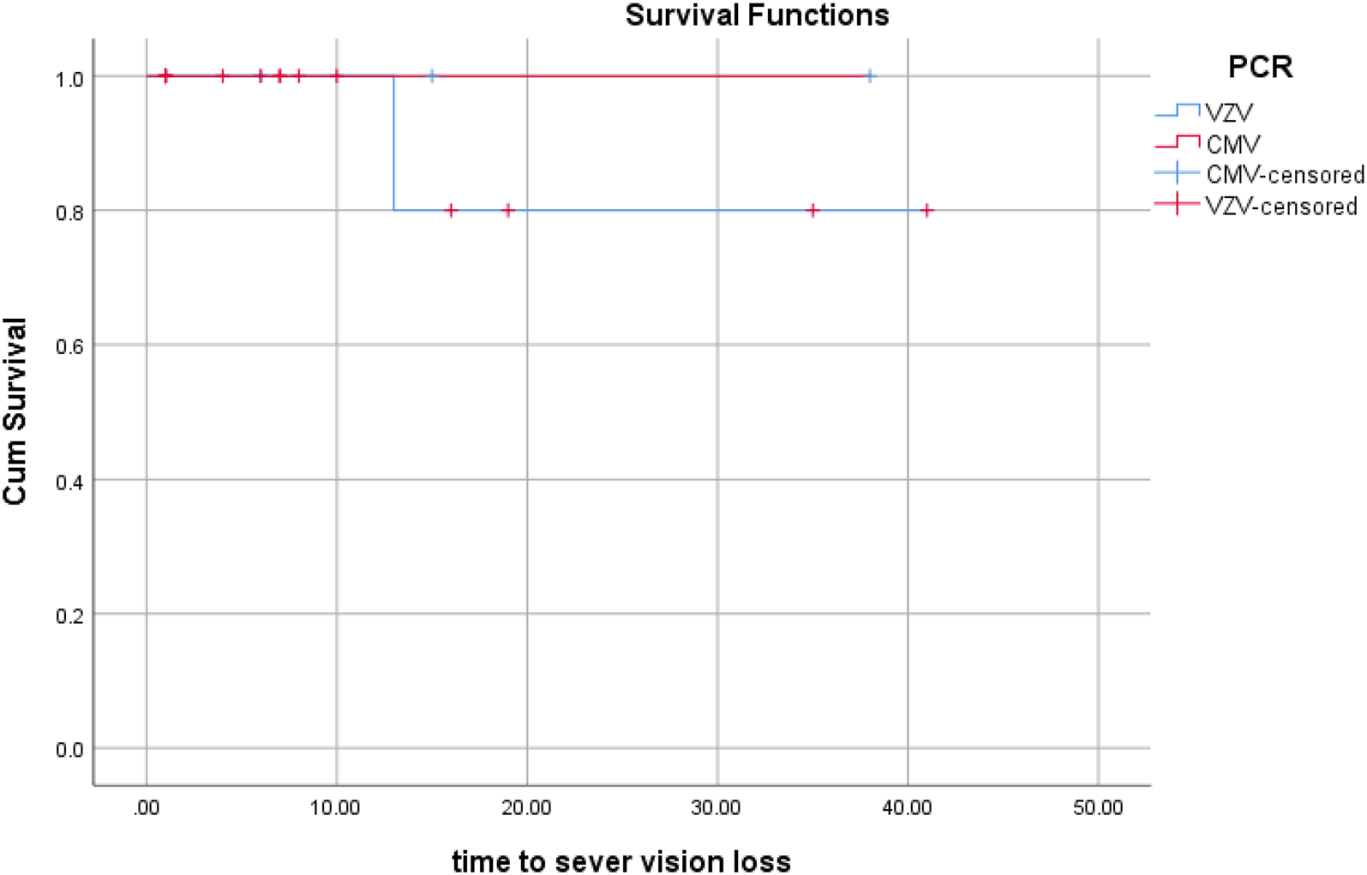
The rate of severe vision loss over time (per month). Kaplan–Meier analysis showed a higher, but not statistically significant, rate in VZV group. Every fall (downward step) in the Blue line means one event (severe VA loss). No event happened in the CMV group

**Fig. 3:**
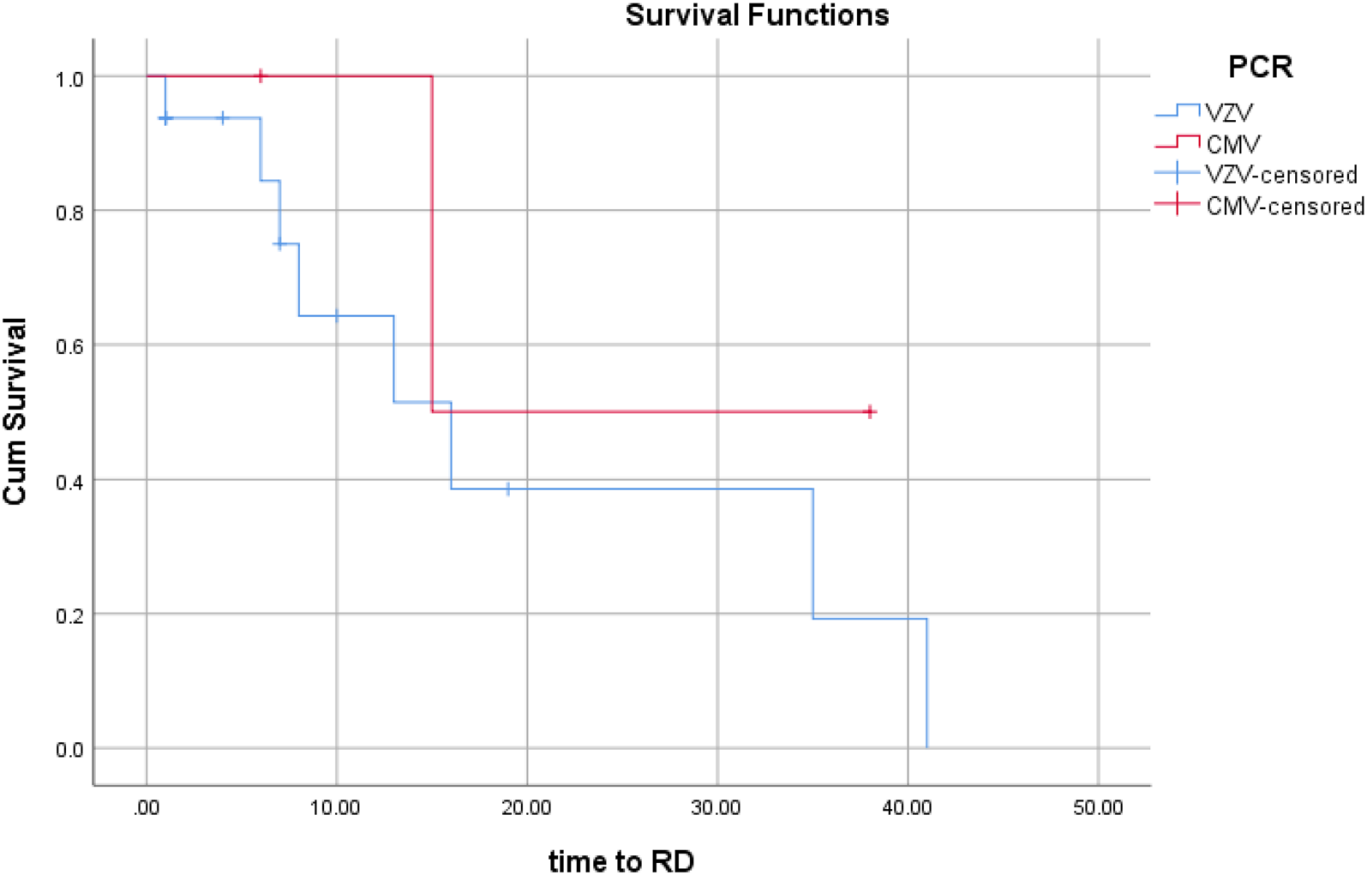
The rate of severe RD over time (per month). Kaplan–Meier analysis showed a higher, but not statistically significant, rate in VZV group. Every fall (downward step) means one event (RD)

Survival curves illustrate the divergence of curves between CMV and VZV for 2-line VA gain, SVL, and RD rates (Figs. 1, 2 and 3, respectively).

## Discussion

The current study showed that the majority of cases (69.6%) had VZV-associated ARN. All patients (100%) were treated with combined systemic and intravitreal antivirals. Several studies have investigated the effectiveness of adjunctive intravitreal foscarnet or ganciclovir and showed promising results in the reduction of severe vision loss (SVL) and RD [17, 18]. However, the intravitreal injection does not prevent the fellow eye involvement and should always be used as an adjunct to systemic therapy [25]. Recent studies have found that intravitreal antiviral, adjuvant to the widely accepted systemic antiviral therapy, improves the visual outcome and reduces the rate of RD or SVL in ARN [9, 18, 26]. Therefore, the use of intravitreal antiviral therapy has increased in the treatment paradigm of ARN.

Despite these treatments, a significant proportion of patients with ARN have poor visual, as well as anatomic outcomes, and end up with RD or SVL. Since there are reports regarding the efficacy of intravitreal treatment [8, 9, 18, 26, 27], this study aimed to evaluate and compare the visual outcome and RD incidence in ARN patients treated with combined intravitreal and systemic antiviral treatment (as the main treatment protocol in our tertiary eye hospital) in the region under study.

In the current study, patients with ARN experienced some improvement in VA with treatment. The most VA improvement occurred 6 months after the initial symptoms but it was not statistically significant. One of the important factors contributing to the poor visual outcomes was the development of RD, which is in line with the findings of previous studies reporting that only 4% of eyes with at least one episode of RD achieved a best corrected VA of ≥ 20/40. [9, 28]

In a case series in 2021, Hedayatfar et al. showed that VZV was the leading cause of ARN (78%) [29]. They reported 61.1% of RD incidence received intravitreal ganciclovir. The present study had a lower rate of RD occurrence (39% vs. 61.1%), a higher intravitreal injection rate (100% vs. 61.1%), and a shorter follow-up, compared to those estimated in the study by Hedayatfar et al. One limitation of the present study is the short follow-up time that may affect the rate of RD or other sight-threatening complications of ARN, such as epiretinal membrane, chronic cystoid macular edema, and the recurrence of inflammation or infection.

In a study by Baltinas et al., despite oral or intravenous therapy with or without intravitreal antiviral therapy, two-thirds of eyes with ARN developed RD [30].

Several other studies have reported the peak incident period of RD within 3–6 months after the initial diagnosis, which affects between 35 and 80% of the involved eyes [17, 20, 31, 32]. In this study, the rate of RD was 39%, and the majority of RD development occurred in the first 3 months (24–307 days) after the disease. In the study by Hedayatfar et al. [29], the rate of RD was 61.1%, which is higher than that in the present study, and the median time for the occurrence of RD was 12 weeks (range 6–25 weeks) after the disease onset, which is comparable to that in the present study.

Recent studies have demonstrated a greater extent of retinitis is associated with the worst visual prognosis [9, 28, 33]. However, there was no significant association between the extent of retinitis (more than two quadrants) with the initial and final VA in the present study. Some of the discrepancies between these findings is due to the retrospective nature of the existing reports, including this study, the diversity in the methods of estimating the cumulative number of involved quadrants, which are mostly based on observation instead of a standard objective method, and the grade of vitreous haziness precluding precise estimation.

Although intravitreal injection carries a small risk of RD, the higher concentration of antiviral medicine, which is directly injected into the main site of inflammation, would halt retinal necrosis progression more efficaciously. However, the number of intravitreal injections in patients with RD was not different from that in non-RD patients (P > 0.05). In a study by Meghpara et al. [9], it was found that the effect of intravitreal ganciclovir injection was limited on VA improvement in patients with extensive retinal necrosis (> 50% of the retinal area). Since most of the cases had more than 50% of retinal involvement, the present results can be compared to this report.

Another principal factor associated with SVL was poor VA at the initial diagnosis [27]. In this study, 14 eyes had an initial VA of better than 1.00 LogMAR (Snellen, 20/200), from which 10 eyes (71%) improved or did not change in VA. From nine eyes with an initial VA of worse than 1.00 LogMAR, six eyes (67%) got worse or did not change in VA. Therefore, the findings showed that poor initial VA was associated with poor visual outcomes.

At the last visit, six out of 23 eyes (26%) maintained a vision of 0.30 LogMAR (Snellen, 20/40) or better, and 10 eyes (43%) had 1.00 LogMAR (Snellen, 20/200) or worse. This result shows that despite aggressive treatment, the visual outcome was poor, yet in accordance with other studies [6, 28].

Similar to other reports [31, 32], the authors found VZV as the leading cause of ARN (69.6%). In addition, 17.3% of cases had a history of prior remote Herpes virus infection, which was lower than other reports [3, 5, 32].

The type of virus is another factor that may have prognostic importance. Previous studies have demonstrated that VZV retinitis occurred in older patients and was associated with a higher SVL rate, in comparison with HSV retinitis [26, 34]. However, the mean age of patients under study in the VZV group (50 ± 9) was lower than that CMV group (60 ± 4).

In the present study, although statistically not significant, the risk of SVL and RD development was higher in VZV-associated ARN (50% and 6.3%, respectively), in comparison with non-VZV-associated ARN (25% and 0%, respectively) (P = 0.80 and P = 0.375, respectively), which is in agreement with previous findings [26, 34]. The reason for failure in reaching a statistically significant level may be the small sample size in the non-VZV group.

In this study, there was no significant difference between the rates of SVL and RD in patients with early treatment (less than 14 days from the beginning of symptoms) and late treatment (more than 14 days after the initial symptoms (P = 0.545 and P = 0.510, respectively). In a study by Khochtali et al., treatment delay for more than 4 weeks was associated with worse visual outcomes but not with RD occurrence [35].

Systemic antiviral is the mainstay of treatment for ARN. Oral valaciclovir was prescribed in different doses ranging from 1000 to 2000 mg per dose three times daily to achieve sufficient vitreous concentration similar to intravenous acyclovir. The efficacy of oral valacyclovir on the resolution of retinitis, decreasing the risk of RD, and improvement of the visual outcome are comparable to intravenous acyclovir [15, 36].

Based on the findings of different studies, visual outcomes, SVL, and RD development are comparable and not statistically significant for ARN patients treated with oral valaciclovir or intravenous acyclovir [30, 36].

Patients under treatment with valaciclovir and acyclovir are at risk of acute kidney injury, and thus, laboratory monitoring and dose adjustment should be considered in chronic kidney disease [37].

The high rate of CMV positivity in this series emphasizes the importance of PCR in the diagnosis and management of ARN. Because CMV retinitis is usually not responsive to acyclovir or valacyclovir and needs to be treated with ganciclovir or valganciclovir treatments.

The other interesting result in the present study is the rarity of HSV-1 and HSV-2 positivity, in contrast to other studies [27, 34].

Eight out of 23 patients (35%) may have had subclinical immune dysfunction, including diabetes mellitus, kidney transplant, malignancy, and chemotherapy. There was no significant difference between the disease severity, as well as the clinical course of these patients, and otherwise healthy patients.

For precise comparison of different types of viruses, further multicenter studies are recommended with a prospective design, a larger sample size, and longer follow-ups.

The present study findings are in line with the findings of previous studies, demonstrating that despite the combination therapy of systemic and intravitreal anti-viral, ARN still carries a poor prognosis with approximately 39.1% vision loss to the level of 1.00 LogMAR (Snellen, 20/200) or worse. [31, 32].

We used oral corticosteroids as an adjuvant therapy in all of our patients. Despite conflicting results about their positive role in final visual acuity (VA), corticosteroids are used in many centers as adjuvant therapy [20, 38, 39].

The use of anticoagulants, such as aspirin, has been suggested; however, there is not enough evidence proving their effectiveness.

The findings of this study are not consistent with previous study findings regarding the association between RD occurrence and SVL, and also, the positive effect of intravitreal antiviral on the final visual outcome. However, these findings could confirm that eyes with RD had poorer VA outcomes and younger patients had more VA improvement.

Although the findings of this study showed the effectiveness of combination therapy with intravitreal antiviral, multiple aspects of the treatment were not evaluated, such as the duration of optimal treatment for immunocompromised patients, the requirement of long-term prophylactic therapy, and the role of corticosteroid therapy in visual outcomes, which warrant further studies.

The most important limitations of this study include its retrospective nature and small sample size. Additionally, the types and times of surgery were not assessed.

## Conclusions

In conclusion, the overall outcomes of ARN were generally disappointing regardless of the early treatment and intravitreal antiviral injection. The findings confirm previous findings claiming that the majority of patients with ARN ended with SVL despite early diagnosis and treatment.

## Data Availability

Not applicable.

## Abbreviations

ARN: Acute retinal necrosis
PCR: Polymerase chain reaction
VA: Visual acuity
RD: Retinal detachment
VZV: Varicella zoster virus
CMV: Cytomegalovirus
HSV: Herpes virus
SVL: Severe vision loss
LogMAR: Logarithm of the minimum angle of resolution
RRD: Rhegmatogenous Retinal Detachment
